# *Salmonella* Typhi-specific multifunctional CD8+ T cells play a dominant role in protection from typhoid fever in humans

**DOI:** 10.1186/s12967-016-0819-7

**Published:** 2016-03-01

**Authors:** Stephanie Fresnay, Monica A. McArthur, Laurence Magder, Thomas C. Darton, Claire Jones, Claire S. Waddington, Christoph J. Blohmke, Brian Angus, Myron M. Levine, Andrew J. Pollard, Marcelo B. Sztein

**Affiliations:** Center for Vaccine Development, University of Maryland School of Medicine, 685 W. Baltimore Street, Suite 480, Baltimore, MD 21201 USA; Department of Epidemiology and Public Health, University of Maryland School of Medicine, Baltimore, MD USA; Oxford Vaccine Group, Department of Paediatrics, University of Oxford and the NIHR Oxford Biomedical Research Centre, Oxford, UK; Nuffield Department of Medicine, University of Oxford, Oxford, UK

**Keywords:** Typhoid fever, *Salmonella* Typhi, Cell-mediated immunity, CMI, CD8 T cells, Multifunctional, Cytotoxicity, Cytokines

## Abstract

**Background:**

Typhoid fever, caused by the human-restricted organism *Salmonella* Typhi (*S.* Typhi), is a major public health problem worldwide. Development of novel vaccines remains imperative, but is hampered by an incomplete understanding of the immune responses that correlate with protection.

**Methods:**

Recently, a controlled human infection model was re-established in which volunteers received ~10^3^ cfu wild-type *S.* Typhi (Quailes strain) orally. Twenty-one volunteers were evaluated for their cell-mediated immune (CMI) responses. Ex vivo PBMC isolated before and up to 1 year after challenge were exposed to three *S.* Typhi-infected targets, i.e., autologous B lymphoblastoid cell-lines (B-LCL), autologous blasts and HLA-E restricted AEH B-LCL cells. CMI responses were evaluated using 14-color multiparametric flow cytometry to detect simultaneously five intracellular cytokines/chemokines (i.e., IL-17A, IL-2, IFN-g, TNF-a and MIP-1b) and a marker of degranulation/cytotoxic activity (CD107a).

**Results:**

Herein we provide the first evidence that *S.* Typhi-specific CD8+ responses correlate with clinical outcome in humans challenged with wild-type *S.* Typhi. Higher multifunctional *S.* Typhi-specific CD8+ baseline responses were associated with protection against typhoid and delayed disease onset. Moreover, following challenge, development of typhoid fever was accompanied by decreases in circulating *S.* Typhi-specific CD8+ T effector/memory (T_EM_) with gut homing potential, suggesting migration to the site(s) of infection. In contrast, protection against disease was associated with low or no changes in circulating *S*. Typhi-specific T_EM_.

**Conclusions:**

These studies provide novel insights into the protective immune responses against typhoid disease that will aid in selection and development of new vaccine candidates.

**Electronic supplementary material:**

The online version of this article (doi:10.1186/s12967-016-0819-7) contains supplementary material, which is available to authorized users.

## Background

Typhoid fever constitutes a major global health problem, with an estimated 21.7 million cases and 200,000 deaths annually [1]. The development of improved vaccines is necessary, but advances have been delayed by a lack of knowledge of the immunological correlates of protection against *Salmonella enterica* serovar Typhi (*S*. Typhi). Since the causative agent of typhoid fever, *S*. Typhi, is a human-restricted bacteria [2], current knowledge is limited due to the difficulties associated with performing challenge studies in humans and the lack of adequate pre-clinical models that closely mimic typhoid fever.

Recently, a human challenge model was established by the Oxford Vaccine Group (OVG, University of Oxford) in which naïve participants ingested wild-type (wt) *S.* Typhi (Quailes strain) [3, 4]. This controlled infection study was modeled after the human typhoid challenge studies performed in the 1960s at the University of Maryland. The Maryland studies improved understanding of typhoid fever [5–8] and resulted in the initiation of the process to license the oral attenuated Ty21a typhoid vaccine [9], but did not identify the immunological correlates of protection. Although substantial data are available on the immune responses after infection in the field or following vaccination, there are no studies that provide insights into the immunological status before wild-type infection and its possible effects on clinical outcome.

The re-establishment of the human challenge model with wt *S.* Typhi, and the use of cutting-edge multichromatic flow cytometry allowed us, for the first time, to investigate the pre-challenge immunological status and its correlation with the subsequent clinical outcome. Furthermore, it allowed the initiation of detailed studies of the kinetics and characteristics of the immunological responses occurring following infection with wt *S.* Typhi.

Several immunization studies with attenuated typhoid vaccine candidates suggested that cell-mediated immunity (CMI), particularly CD8+ effector T cells, constitute a major component in the control of typhoid fever [10, 11]. CD8+ T cells may be involved in destroying infected-host cells through cytolytic activity and/or production of cytokines (e.g., interferon (IFN)-γ, tumor necrosis factor (TNF)-α, interleukin (IL)-17) [12–22].

Recent research on the immune responses after oral immunization with Ty21a have revealed persistent multiphasic, multifunctional (simultaneous production of multiple cytokines) responses to antigenic presentation by class Ia HLA and by the more conserved and less polymorphic non-classical HLA-E molecules [13, 19, 20, 22].

In the present study we investigated the relationship between *S*. Typhi-specific CD8+ T cell responses before exposure to wt *S.* Typhi and clinical outcome, i.e., whether the participants who were challenged developed disease or not. We also explored *S*. Typhi-specific CD8+ T cells responses following challenge, as well as their gut homing potential in relationship with typhoid diagnosis. Finally, we identified the dominant multifunctional S. Typhi-specific response patterns associated with clinical outcome by evaluating the simultaneous production of macrophage inflammatory protein (MIP)-1β, IFN-γ, TNF-α, IL-2 and IL-17A, as well as the expression of the cytotoxicity degranulation marker CD107a [23]. These investigations provide evidence that baseline *S.* Typhi-specific responses are related to clinical outcome after wt *S.* Typhi infection and provide novel insights into the immunological responses involved in protection following natural infection and vaccination.

## Methods

### Participants and study design

Twenty-one healthy, male or female participants aged 18–60 years were recruited by the Oxford vaccine Group, Department of Paediatrics, Oxford, UK, to participate in this dose-escalation study. Any participant with a history of typhoid fever or immunization against typhoid fever, or who lived in a typhoid-endemic region for longer than 6 months, was excluded from participation. Only participants with low risk of becoming chronic carriers (including those without gall stones, determined by ultrasound examination of the gallbladder) were included. Participants were challenged orally with a dose of 1–5 × 10^3^ CFU of wt S. Typhi (Quailes strain) suspended in sodium bicarbonate. The *S.* Typhi Quailes strain, which was used extensively for human challenge studies in the 1960s/1970s was developed by the University of Maryland and used to establish a master cell bank in Oxford. Participants were monitored closely throughout the study. A positive typhoid fever diagnosis was defined based on the following criteria: either a positive blood culture for *Salmonella *Typhi from day 5 post-challenge or, oral temperature ≥38 °C, persisting continuously for at least 12-h in the absence of anti-pyretic medication, occurring from 72-h after challenge. At the point of typhoid fever diagnosis (TD, as determined by *S.* Typhi bacteremia or development of a fever >38 °C for ≥12 h) participants were treated with a 2-week course of antibiotics (ciprofloxacin, 500 mg BD). Participants who did not developed typhoid fever (NoTD) received a 2-week course of antibiotics at day 14 post-challenge. Peripheral blood mononuclear cells (PBMC) obtained from 16 selected participants based on cell availability were analyzed in these studies (Additional file 1: Table S1). These included all nine who were not diagnosed with typhoid (NoTD) and seven participants were diagnosed with typhoid (TD).

### Ethics statement

Written informed consent was obtained and all procedures approved by National Research Ethic Service (NRES), Oxfordshire Research Ethics Comittee A (10/H0604/53) and conducted in accordance with the principles of the International Conference of Harmonisation Good Clinical Practice guidelines.

### Specimen collection and isolation of PBMC

Blood was collected from each participant and routine blood hematology was assessed on alternate days after challenge and at typhoid diagnosis. PBMC were separated by Lymphoprep gradient centrifugation (Axis-Shield, Oslo, Norway) cryopreserved in liquid nitrogen following standard techniques within 4 h of initial blood draw within 4 h of initial blood draw and stored in liquid N_2_. Upon thawing, viability and recovery were measured using trypan blue exclusion and a Guava easyCyte™ flow cytometer (Merck KGaA, Darmstadt, Germany), and cells were rested overnight in complete RPMI (cRPMI: RPMI 1640 media (Gibco, Carlsbad, CA) supplemented with 100 U/mL penicillin (Sigma), 100 µg/mL streptomycin (Sigma), 50 μg/mL gentamicin (Gibco), 2 mM l-glutamine (Gibco), 10 mM HEPES buffer (Gibco) and 10 % heat-inactivated fetal bovine serum (Gemini Bioproducts, West Sacramento, CA) to serve as effector cells in CMI assays.

### Stimulator cells

Autologous Epstein-Barr virus (EBV)-transformed lymphoblastoid cell line (B-EBV cells) and autologous blasts were generated from the PBMC of each participant isolated before challenge. B-EBV cells were obtained by incubation of the PBMC with EBV-containing supernatant from the B95-8 cell line (ATCC CRL1612) and cyclosporine (0.5 ug/mL; Sigma-Aldrich, Saint-Louis, MO) at 37 °C with 5 % CO_2_ for 2–3 weeks. PHA-activated blasts were prepared by incubating PBMC with 1 μg/ml PHA (Sigma-Aldrich, St. Louis, MO) in cRPMI for 24 h, followed by washing and culture in cRPMI containing 20 IU/ml recombinant human IL-2 (rhIL-2; Roche, Indianapolis, IN) for 7 days. The HLA classical class I-defective B cell line 721.222.AEH which expresses non-classical class-I HLA-E molecules was provided by Dr. D. Geraghty [14] and cultured in cRPMI supplemented with 200 mU/ml hygromycin B (Sigma-Aldrich).

### *S*. Typhi infection of stimulator cells

Stimulating cells were infected by incubation for 3 h at 37 °C with wt-*S.* Typhi strain ISP1820 at a multiplicity of infection of 7:1 in RPMI without antibiotics, washed three times with cRMPI and incubated overnight with cRPMI containing 150 μg/ml gentamicin. Cells were washed and infection with *S.* Typhi was confirmed by staining with anti-*Salmonella* common structural Ag (CSA-1) (Kierkegaard and Perry, Gaithersburg, MD) and analysis by flow cytometry as previously described [10].

### Ex-vivo stimulation of effector cells

PBMC were thawed and rested in cRPMI overnight at 37 °C in 5 % CO_2_ before stimulation with S. Typhi-infected stimulating cells. Uninfected target cells and Staphylococcal enterotoxin B (SEB; 10 μg/ml) were used as negative and positive controls, respectively. Target cells were gamma-irradiated (6000 rad) and co-cultured with PBMC (effector:stimulator ratio 5:1) in the presence of the FITC-conjugated anti-CD107a (BD Biosciences) monoclonal antibody (mAb). After 2 h, the protein transport blockers monensin (1 µg/ml, Sigma) and brefeldin A (2 µg/ml; Sigma) were added to the PBMC and cells were incubated overnight at 37 °C in 5 % CO_2_. For the kinetics analysis, because of the variable responsiveness at baseline in the different participants, the net values (day x post-challenge—day 0) were used to normalize the data.

### Immunostaining with 14-color panel mAbs

Following stimulation, PBMC were harvested, washed in 1X PBS and stained for dead-cell discrimination using Yellow Live/Dead viability kit (Invitrogen, Carlsbad, CA). Cells were then washed with wash buffer (PBS 1 % FCS) and non-specific Fc receptor binding was blocked with human immunoglobulin (3 µg/mL; Sigma) for 10 min at room temperature (RT). Cells were surface stained with mAbs against CD14-BV570 (M5E2, Biolegend), CD19-BV570 (HIB19, Biolegend), CD3-BV650 (OKT3, Biolegend), CD4-PE-Cy5 (RPA-T4, BD), CD8-PerCP-Cy5.5 (SK1, BD), CD45RA-biotin (HI100, BD), CD62L-APC-A780 (DREG-56, Ebioscience) and integrin α_4_β_7_-A647 (ACT1; conjugated in house) at 4 °C for 30 min. Cells were washed with wash buffer and stained with streptavidin (SAV)-Qdot800 (Invitrogen) at 4 °C for 30 min. Cells were then fixed and permeabilized with Fix and Perm buffers (Invitrogen). Intracellular staining was then performed with mAbs against CD69-ECD (TP1.55.3, Beckman Coulter), IFN-γ-PE-Cy7 (B27, BD), TNF-α-A700 (MAb11, BD), IL-2-BV605 (MQ1-17H12, Biolegend), IL-17A-BV421 (BL168, Biolegend) and MIP-1β-PE (IC271P, R&D) at 4 °C for 30 min. Cells were washed with PBS 1 % FCS, fixed in 1 % paraformaldehyde (PFA) and stored at 4 °C until analyzed. Samples were acquired by flow cytometry using a customized LSRII flow cytometer (BD Biosciences) and analyzed using Winlist v7.0 (Verity Software House, Topsham, ME). CD8+ T cells were selected after a gating strategy involving the exclusion of dead cells and CD3−, CD14+ and CD19+ cells. Absolute numbers of CD3 +, CD8+, CD4+ cells and CD8+ memory subsets cells were calculated by using percentages obtained from flow cytometry analysis related to the absolute number of lymphocytes determined by routine blood count. *S.* Typhi-specific responses were expressed as net percentage of positive cells (background after stimulation with uninfected cells were subtracted from values obtained with *S.* Typhi-infected stimulators).

### Statistical analyses

Mann–Whitney tests and linear regression analysis were performed using Prism v7.02 (Graphpad software, La Jolla, CA). Areas under the curve were measured using the trapezoidal method (GraphPad Prism v7.02). P values <0.05 were considered significant. Some comparisons were based on multiple outcomes from the same individual as indicated in the text. In these cases the same individual provided information on cytokine production and/or CD107a expression levels after stimulation with three types of stimulations (EBV-B, AEH, and blasts), and these responses we evaluated with regard to clinical outcome (i.e., TD vs. NoTD patients). In these analyses we accounted for the correlation between multiple outcomes from the same person using a mixed effects model fit by maximum likelihood, including a random effect for person, using SAS 9.3 (Cary, NC). We found by performing simulations that this correlation model provided more accurate statistical inference than models with more complex correlation structure given the small sample sizes.

## Results

### Baseline *S.* Typhi-specific CD8+ T cell responses correlate with clinical outcome after challenge

CD8+ T cells have been reported to be a major component of the CMI response against *S.* Typhi [11–13, 16, 22]. Thus, we first explored whether CD8+ T cell responses in healthy participants at baseline could predict clinical outcome after challenge with 1–5 × 10^3^ CFU of wt *S.* Typhi. All nine volunteers who did not develop typhoid disease (NoTD group) and seven participants who did develop typhoid disease (TD group) were evaluated. Peripheral blood mononuclear cells (PBMC) isolated from volunteers before challenge (day 0) were stimulated in vitro with *S.* Typhi-infected autologous B-EBV, *S.* Typhi-infected autologous blasts or *S.* Typhi-infected AEH cells (the latter to measure HLA-E-restricted CMI). PBMC were evaluated using 14-color multiparametric flow cytometry and CD8+ T cells were divided based on their expression of CD62L and CD45RA into naïve T (TN; CD62L+CD45RA+), T central memory (T_CM_; CD62L+CD45RA−), T effector memory (T_EM_; CD62L−CD45RA−), and T effector memory CD45RA+ (T_EMRA_; CD62L−CD45RA+) subsets. No significant differences were observed at baseline between the TD and NoTD groups in the absolute number of white cells, lymphocytes, CD3, CD8 and CD4 memory subsets (Additional file 1: Figure S1). *S.* Typhi-specific responses were further characterized by co-expression of the T cell activation marker CD69 and the cytotoxicity degranulation marker CD107a, as well as the production of IFN-γ, TNF-α, MIP-1β, IL-17A and IL-2.

**Fig. 1:**
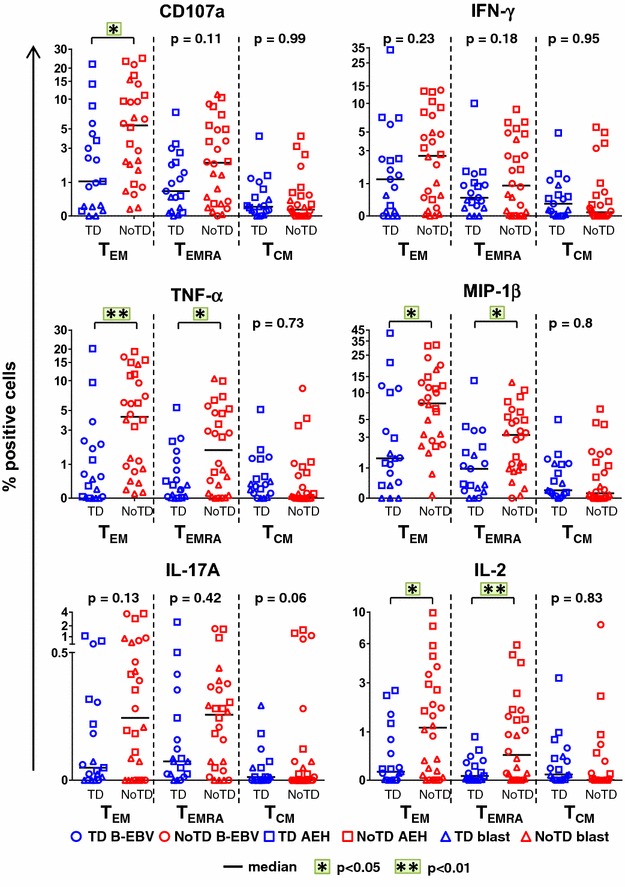
Baseline *S.* Typhi-specific CD8+ T cell responses define the clinical outcome after challenge. PBMC isolated at baseline from each participant (TD n = 7, *blue*; NoTD n = 9, *red*) were stimulated for 18 h with *S.* Typhi-infected AEH cells (*squares*), *S.* Typhi-infected B-EBV cells (*circles*) or *S.* Typhi-infected blasts (*triangles*). After co-culture, cells were immunostained with a 14-color panel of mAbs and analyzed by follow cytometry as described in Methods. Each symbol represents the net percentage of positive cells measured for CD107a, IFN-γ, TNF-α, MIP-1β, IL-17A and IL-2 in the CD8+ T_EM_, T_EMRA_ and T_CM_ subsets as indicated for each participant. Horizontal lines represent the median for each group. Statistical analyses were performed using mixed effects models to account for multiple observations per person. *p < 0.05, **p < 0.01

Most participants who did not develop infection (NoTD) showed higher *S.* Typhi-specific T_EM_ and T_EMRA_ responses at baseline than participants diagnosed with typhoid disease (TD) (Fig. 1). Since these differences were observed after stimulation with the different *S.* Typhi-infected cells, we combined them for the analyses shown in Fig. 1. Consistent with our previous findings [17, 19, 20, 24], T_EM_ cells (T_EM_ and T_EMRA_) represented the major subsets exhibiting intracellular chemokine/cytokine production. Significant differences were observed between the TD and NoTD groups for the expression of CD107a in T_EM_, as well as the production of TNF-α, MIP-1β and IL-2 in T_EM_ and T_EMRA_. Similar trends, albeit not significant, were also observed in CD107a expression and IFN-γ production by T_EMRA_ and IL-17A production by T_EM_, T_EMRA_ and T_CM_. No differences were observed in the T_CM_ subset, except for IL-17A production but this difference did not reach significance.

**Fig. 2:**
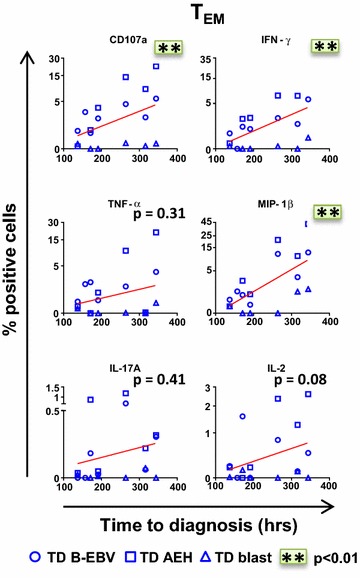
High levels of *S.* Typhi-specific CD8+ T cell responses are associated with delayed time to diagnosis. After stimulation of PBMC with *S.* Typhi-infected AEH cells (*squares*), *S.* Typhi-infected B-EBV cells (*circles*) or *S.* Typhi-infected blasts (*triangles*), the percentages of CD107a, IFN-γ, TNF-α, MIP-1β, IL-17A and IL-2 positive CD8+ T_EM_ cells were plotted against time to diagnosis for each participant who developed typhoid fever (n = 7). Correlations (*red lines*) were assessed using the linear regression function and performed using mixed effects models to account for multiple observations per person. *p < 0.05; **p < 0.01

Interestingly, among the volunteers diagnosed with typhoid, we also found a positive relationship (significant in many cases) between *S.* Typhi-specific baseline levels of all markers in T_EM_ and delay in time of disease onset (Fig. 2). These relationships were significant for CD107a expression, and IFN-γ and MIP-1β production. These data show that the presence of higher levels of *S*. Typhi-specific T cells before challenge were associated with protection against typhoid fever and delayed time to disease onset.

### Distinct clinical outcomes are accompanied by discrete *S.* Typhi-specific responses after challenge

**Fig. 3:**
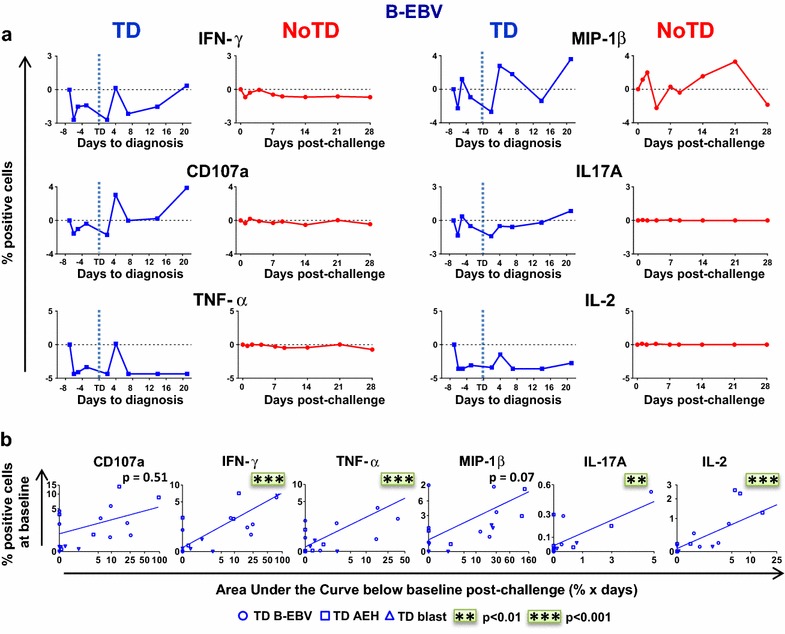
Post-challenge kinetics of *S.* Typhi-specific CD8+ T cell responses differs with clinical outcome. **a** Kinetics for a representative participant from each group (TD and NoTD) showing the production of cytokines/chemokines and expression of CD107a by CD8+ T_EM_ following stimulation with *S.* Typhi-infected B-EBV cells at different time points after challenge. **b** Areas under the curve below baseline were measured until time of diagnosis and* plotted* against the baseline level of responses for each of the indicated biomarkers stimulated with *S.* Typhi-infected AEH cells (*squares*), *S.* Typhi-infected B-EBV cells (*circles*) or *S.* Typhi-infected blasts (*triangles*). Correlation analyses were performed using mixed effects models to account for multiple observations per person. *p < 0.05, **p < 0.01, ***p < 0.001

After identifying correlations between baseline responses and clinical outcome, we examined the kinetics of immune responses until day 28 after challenge. Decreases in the absolute numbers of CD3+, CD8+ were observed in TD participants before diagnosis (Additional file 1: Figure S2A), a finding consistent with the decreased lymphocyte counts previously reported [3]. Decreases in the absolute numbers of *S.* Typhi-specific T_EM_ expressing CD107a and cytokine-producing cells were also observed (Additional file 1: Figure S2B) in TD subjects. In contrast, these decreases in the absolute numbers of *S.* Typhi-specific T_EM_ expressing CD107a or producing IFN-γ were not observed in NoTD participants (Additional file 1: Figure S2B). We also investigated whether challenge with wt *S.* Typhi elicited multiphasic responses such as those observed after vaccination with Ty21a [19, 20]. Soon following challenge and before the onset of disease, 5 out of 6 TD evaluable participants showed a decrease in circulating CD107a-expressing and cytokine-producing T_EM_ cells following stimulation with *S.* Typhi-infected B-EBV cells (Fig. 3a, Additional file 1: Figure S3A). In most TD subjects, this early drop was followed by a sharp rebound above baseline levels after disease onset. In contrast, no changes were observed in 4 out of the 6 NoTD participants (Additional file 1: Figure S3A). Of note, in three NoTD participants who did not reach the criteria for typhoid disease diagnosis, *S.* Typhi was detected either in blood by PCR or in stool (Additional file 1: Figure S3A). Interestingly, these NoTD volunteers showed similar decreases after challenge to those observed in TD volunteers. Of note, variability in the magnitude of the decrease after challenge was noticed among TD participants (Additional file 1: Figure S3A). We identified a positive trend for all biomarkers between the magnitude of the decrease below baseline (area under the curve (AUC)) and their baseline levels. Significant correlations in these measurements were observed for IFN-γ, TNF-α, IL-17A and IL-2 in T_EM_ cells (Fig. 3b). Notably, T_EM_ cells showed a more pronounced decrease for all makers, followed by T_EMRA_ and T_CM_ cells (Additional file 1: Figure S3B).

### Enhanced gut homing potential of *S.* Typhi-specific CD8+ T cells in participants diagnosed with typhoid disease

**Fig. 4:**
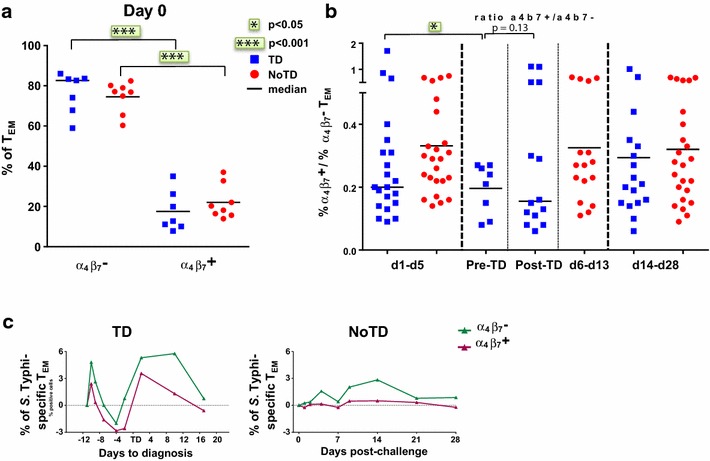
Gut homing potential of total CD8+ T_EM_ and *S.* Typhi-specific CD8+ T_EM_. **a** Each value represents the baseline percentages of integrin α_4_β_7_− and integrin α_4_β_7_+ cells in total CD8+ T_EM_ evaluated by surface staining and flow cytometry in TD (*blue squares*) and NoTD (*red circles*) participants. *Horizontal lines* represent the median for each group. **p < 0.01; ***p < 0.001. **b** Ratios of integrin α_4_β_7_+ to integrin α_4_β_7_− in total CD8+ T_EM_ cells are shown within three periods of time after challenge as follows: d1-5 represents the period prior to any typhoid diagnosis in all volunteers; d6-d13 in NoTD volunteers corresponds to the time period in which typhoid was diagnosed in the TD subjects; pre-TD and post-TD in TD participants corresponds to day 6 to the day of diagnosis (Pre-TD) and from the day of diagnosis to day 13 (Post-TD); and d14-d28 (the final time segment) includes the specimens collected after the initiation of antibiotic treatment in all volunteers. *Horizontal lines* represent the median for each group. Statistical analyses were performed using mixed effects models to account for multiple observations per person. *p < 0.05. **c** Post-challenge kinetics of the percentages of integrin α_4_β_7_− and integrin α_4_β_7_+ *S.* Typhi-specific CD8+ T_EM_ following stimulation with *S.* Typhi-infected cells B-EBV cells are shown for participants from each group

The mucosal immunity elicited at the site of entry for *S.* Typhi is expected to be a key factor in protection against typhoid fever after vaccination with live oral typhoid vaccines [14, 17]. Therefore, we assessed whether the decrease in *S.* Typhi-specific responses after challenge were at least in part due to the selective migration of CD8+ T cells to the small intestine, as determined by the expression of the gut homing molecule integrin α_4_β_7_ (Fig. 4a) in *S.typhi*-specific T_EM_ cells. We found no difference in the baseline proportions of integrin α_4_β_7_-expressing cells between the TD and NoTD groups in total CD8+ T_EM_ cells (Fig. 4a). Interestingly, a substantial decline in the proportion of integrin α_4_β_7_+ CD8+ T_EM_ was observed in circulation around the time of diagnosis (d6-d14) for TD participants, while this proportion remained unchanged for NoTD participants (Fig. 4b). We next measured the *S.typhi*-specific expression of integrin α_4_β_7_ on activated (CD69+) CD8+ T_EM_ after stimulation with *S.typhi*-infected cells (AEH cells, B-EBV cells or blasts). Early after challenge, TD participants showed similar decreases in both integrin α_4_β_7_− and integrin α_4_β_7_+ *S.typhi*-specific T_EM_, and both populations rebounded over baseline levels after disease diagnosis (Fig. 4c). In contrast, no changes were observed in NoTD participants (Fig. 4c).

### *S.* Typhi-specific CD8+ T_M_ cells are primarily multifunctional (MF)

**Fig. 5:**
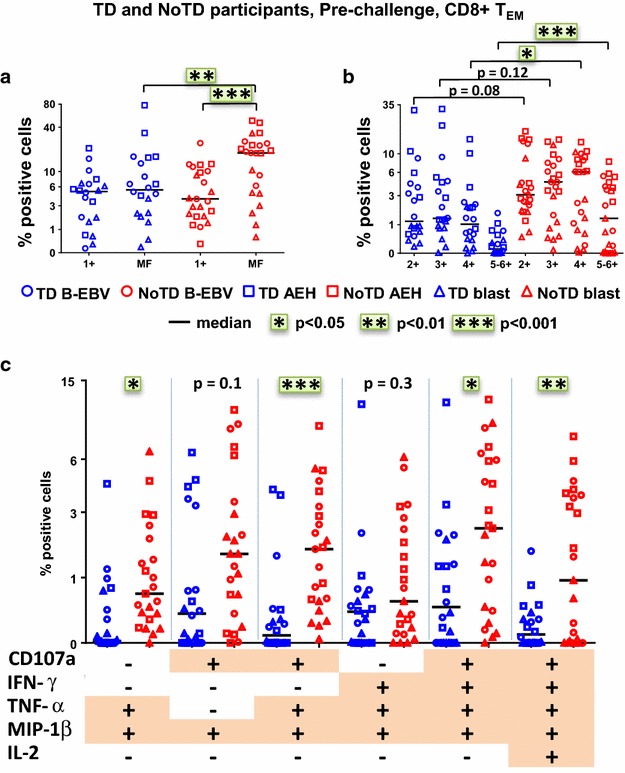
Characterization of multifunctional (MF) *S.* Typhi-specific CD8+ T_EM_ responses at baseline. Flow cytometry data were analyzed using the FCOM function of Winlist to determine the proportion of all possible combinations of the six measured biomarkers to identify MF cells (i.e., concomitantly positive for two or more biomarkers). Each symbol represents the percentage of the different populations measured after stimulation with *S.* Typhi-infected cells (AEH cells [*squares*], B-EBV [*circles*] cells or blasts [*triangles*]) for each participant. **a** The percentages of single positive cells (1+) or of total MF CD8+ T_EM_ cells (i.e., the sum of all cells concomitantly positive for two or more biomarkers). **b** MF cells were divided into four groups on the basis of the number of biomarkers they expressed (e.g., cells expressing two biomarkers are shown as double positive (2+), etc.). **c** Characterization of the six major individual populations of MF in CD8+ T_EM_. Horizontal lines represent the median for each group. Statistical analyses were performed using mixed effects models to account for multiple observations per person. *p < 0.05, **p < 0.01, ***p < 0.001

Concomitant production of cytokines/chemokines and/or expression of CD107a by single cells (multifunctional cells) appear to be central for protective immune responses [19, 20, 25–28]. Thus, we next studied the characteristics of all 64 possible combinations of the six functional biomarkers measured in CD8+ T_EM_ cells at baseline. We first grouped them into single positive (1+) or multifunctional (MF) cells, i.e., cells positive for two or more biomarkers (Fig. 5a). Notably, at baseline, the MF populations were significantly higher in participants who did not develop typhoid disease. We further categorized the MF populations based on the number of biomarkers they exhibited, and observed that double (2+), triple (3+) and quadruple (4+) positive populations comprise the dominant populations of *S.* Typhi-specific MF CD8+ T_EM_ in TD and NoTD participants (Fig. 5b). Moreover, we observed that quadruple (4+) and quintuple-sextuple (5–6+) positive MF cells were significantly higher in the NoTD group, while strong trends were also observed for double and triple positive cells. We further characterized the individual MF populations by identifying the six dominant (i.e., highest frequency) MF populations, which together represented 65–85 % of the total *S.* Typhi-specific MF cells in the NoTD participants (Fig. 5c). The production of the chemokine MIP-1β was a major characteristic in all populations, followed by CD107a expression and production of IFN-γ and TNF-α while IL-2 was detected at considerably lower frequencies. The CD107a+IFN-γ+TNF-α+MIP-1β+, CD107a+TNF-α+MIP-1β+ and CD107a+MIP-1β+ populations were predominant and significantly higher in the NoTD participants. IL-17A was detected at lower levels, and was not produced by any of the 6 dominant populations. Of note, several of the dominant MF populations (e.g., CD107a+MIP-1β+) showed significant correlations with delayed time to disease diagnosis. The MF cells were similarly dominant in both integrin α_4_β_7_− and integrin α_4_β_7_+ T_EM_ in NoTD participants (Additional file 1: Figure S4A, B), however, the TNF-α+MIP-1β+ and CD107+TNF-α+MIP-1β+ populations were significantly higher in integrin α_4_β_7_− T_EM_ subsets (Additional file 1: Figure S4C). No differences were observed between integrin α_4_β_7_− T_EM_ and integrin α_4_β_7_+ T_EM_ in TD participants.

**Fig. 6:**
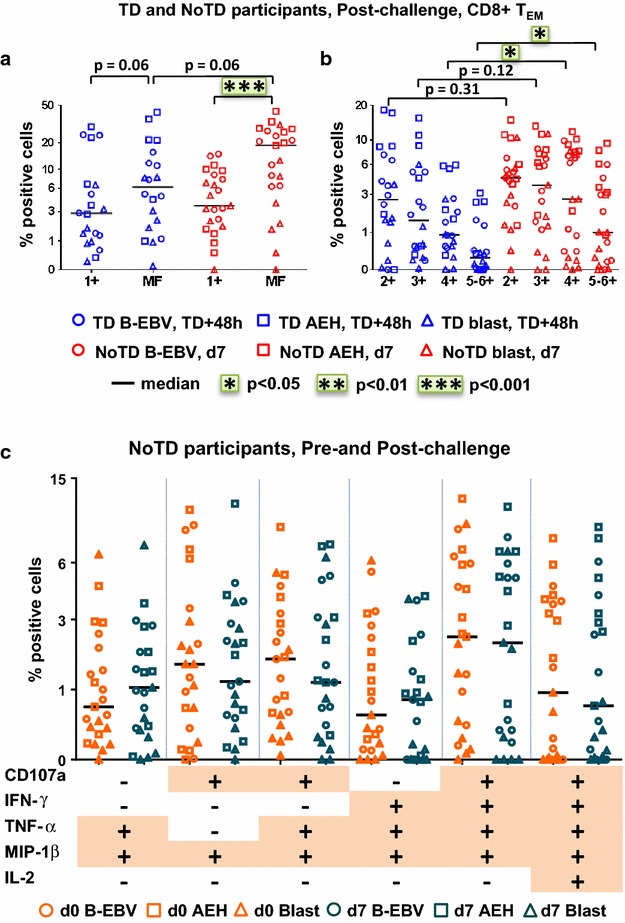
Characterization of multifunctional (MF) *S.* Typhi-specific CD8+ T_EM_ responses after challenge. Flow cytometry data were analyzed as described in Fig. 5. Percentages were measured at day 7 for NoTD participants (d7) and at 48 h after typhoid diagnosis (TD + 48 h) for TD participants. **a** Shown are the percentages of single positive cells (1+) or of total MF cells. **b** Total MF cells were divided into four groups on the basis of the number of biomarkers they expressed. **c** The six major individual populations of MF in NoTD participants are represented at baseline (*orange*) or 7 days after the challenge (*dark green*). *Horizontal lines* represent the median for each group. Statistical analyses were performed using mixed effects models to account for multiple observations per person. *p < 0.05, **p < 0.01, ***p < 0.001

Finally, we observed that *S*. Typhi-specific MF responses were also prevalent at day 7 post challenge in NoTD participants (Fig. 6a, b), and no differences were found in the percentages of the six dominant populations compared to baseline (Fig. 6c). Interestingly, in contrast to what we observed before challenge, in TD subjects we recorded a strong trend towards a dominance of MF cells 48 h after typhoid diagnosis (Fig. 6a). This dominance of MF populations in TD participants at 48 h was more evident in integrin α_4_β_7_+ than in integrin α_4_β_7_− *S.* Typhi-specific T_EM_ subsets (Additional file 1: Figure S5A, B). TNF-α+MIP-1β+, CD107+MIP-1β+ and CD107a+IFN-γ+TNF-α+MIP-1β+ populations in NoTD participants were significantly higher in the integrin α_4_β_7_− T_EM_ subsets (Additional file 1: Figure S5C). However, no differences were observed between integrin α_4_β_7_− T_EM_ and integrin α_4_β_7_+ T_EM_ in TD participants. Taken together, these results point to the importance of MF *S.* Typhi-specific CD8+ T_EM_ with gut, as well as extra-intestinal tissue homing potential in protection from disease.

## Discussion

Studies on typhoid fever immunity in humans have been largely restricted to patients residing in endemic areas and subjects immunized with licensed and experimental typhoid vaccines. In contrast, virtually no information is available on the immune status, particularly regarding CMI, prior to infection and on the immunological correlates of protection following oral exposure to wt *S.* Typhi. The re-establishment of the human challenge model with wt *S.* Typhi provided a unique opportunity to directly assess the impact of *S.* Typhi-specific CMI responses before pathogen exposure to the subsequent development of, or protection from, typhoid disease. Herein we show that higher *S.* Typhi-specific baseline responses are associated with protection against typhoid fever and delayed time to diagnosis and characterized these responses in great detail. If confirmed by future studies involving additional volunteers, these novel observations suggest an important role of anti-*S*. Typhi specific multifunctional CMI responses in protection from disease. This information has the potential to greatly accelerate the development of novel new generation typhoid vaccines.

It has been recently described that the multifunctional quality of the responses is critical for protection against pathogens [23, 25–28]. In the current study we provide evidence that *S.* Typhi-specific MF CD8+ T_EM_ and T_EMRA_ cells are the major effector subsets associated with protection against typhoid fever, as well as delayed time to disease onset. Of note, while MF *S.* Typhi-specific CD8+ cells in NoTD participants were dominant both before and after challenge, this dominance was seen in TD participants only after challenge. MIP-1β was shown to play a role in CTL activity and in controlling infection in HIV non-progressors [29–31]. We previously described the co-production of MIP-1β with IFN-γ, TNF-α, IL-17A and IL-2 following vaccination with Ty21a [20] and MIP-1β was reported to be produced by PBMC obtained from *S.* Typhi-infected convalescent patients [32]. Herein, we show that production of MIP-1β is a major feature of *S.* Typhi-specific MF populations suggesting that co-production of MIP-1β with other cytokines is a key component in protection against *S.* Typhi. These results highlight that a strong MF component of CD8+ T_EM_ responses both at baseline and after challenge, is associated with disease protection and delayed time to diagnosis.

Following challenge we observed decreases in the percentages of circulating *S.* Typhi-specific T_EM_ cells in TD participants, suggesting that these cells might be leaving the systemic circulation during the incubation phase of the disease. Similar to the decreases in lymphocyte counts reported post-challenge in TD participants [3], we also observed a decline in the absolute numbers of CD3+, CD8+ and CD4+ T cells. Integrin α_4_β_7_ expression plays an essential role in the selective homing of T cells to the gut, the site of entry for *S.* Typhi [16, 33–36]. After challenge, TD participants show a decrease in total integrin α_4_β_7_+ CD8+ T_EM_ and in both integrin α_4_β_7_− and α_4_β_7_+ *S.* Typhi-specific T_EM_ cells. However, 48 h after diagnosis, the proportion of *S.* Typhi-specific MF integrin α_4_β_7_+ T_EM_ in TD subjects was higher than in integrin α_4_β_7_− cells. This shift in the balance of single-positive vs. MF *S.* Typhi-specific CD8+ T_EM_ in circulation might be the result of activation and expansion of MF *S.* Typhi-specific integrin α_4_β_7_+ CD8+ T_EM_ cells due to the ongoing infection in the gut and other lymphoid tissues. Taken together, these results highlight that strong MF CD8+ T_EM_ responses with the potential to home to the gut as well as to other lymphoid tissues are associated with disease protection and delayed time to diagnosis. These results complement and expand our previous findings showing that vaccination against *S.* Typhi elicited integrin α_4_β_7_+ and integrin α_4_β_7_− CD8+ effector T cells [14, 16, 17, 24]. These observations also suggest that *S.typhi*-specific T_EM_ cells migrate not only to mucosal sites, but also presumably to secondary lymphoid tissues, where they may reduce *S.typhi* replication during the incubation phase, delaying disease onset. Moreover, post-challenge decreases in circulating *S.* Typhi-specific T cells are proportional to the levels present at baseline, suggesting that the higher the pool of *S*. Typhi-specific T cells available, the higher the number of these cells that are recruited to the sites of inflammation.

Several studies in the murine *S.typhimurium* model have shown that the balance of suppressive regulatory T cell (T_reg_) and pro-inflammatory T cell responses influence bacterial clearance or persistence [37]. We have recently evaluated in these challenged volunteers the hypothesis that the development of T_reg_ responses [38] following exposure to wt *S.* Typhi could be responsible, at least in part, for the decrease of *S.* Typhi-specific T_EM_ responses. We observed that TD participants exhibited up-regulation of the gut homing molecule integrin α_4_β_7_ pre-challenge, followed by a significant down-regulation post-challenge consistent with T_reg_ homing to the gut, as well as up-regulation of activation molecules post-challenge. We also showed that depletion of T_reg_ results in increased *S.* Typhi-specific cytokine production by CD8+ T_EM_ in vitro. These observations suggest that the tissue distribution of activated T_reg_, their characteristics and activation status may play a key role in typhoid fever, possibly through suppression of *S*. Typhi-specific effector T cell responses [39].

In contrast to the results seen in TD participants, we observed that protection against typhoid fever is mostly associated with very low or no changes in circulating *S.* Typhi-specific T_M_ after challenge. Of note, several of the few NoTD participants showing decreases in *S.* Typhi-specific T_EM_ were PCR positive or stool positive participants despite the fact that they did not meet typhoid disease diagnosis. These findings suggest that the control and clearance of *S.* Typhi in NoTD participants might be driven by innate and/or adaptive immune responses in the gut microenvironment, precluding *S.* Typhi from becoming invasive and causing disease. However, the underlying mechanism(s) of control of *S.* Typhi infection in the gut microenvironment remain unclear. “Innate-like” T cell types such as TCRγ/δ T cells, NK-T cells and mucosal associated invariant T (MAIT) cells might be involved in protection from typhoid disease. MAIT cells are comprised of CD8+CD4− and CD8−CD4− subsets restricted by the MR1 MHC-related molecule which are widely believed to play an important in mucosal immunity [40]. We have recently shown that MAIT cells from healthy individuals are able to produce IL-17A, IFN-γ and TNF-α when exposed to *S.* Typhi-infected cells [41]. Ongoing experiments directed to characterize MAIT cell responses in the challenged participants are expected to shed light into the role of these cells following exposure to wt *S.* Typhi.

Because of the very limited data available regarding immune responses prior to typhoid infection, the reasons for the disparity in baseline responses observed among the participants are unclear. Participants were recruited in the UK, a non-endemic region, have not been vaccinated against typhoid, and therefore have likely never encountered *S.* Typhi. However, differences in baseline responses could be due to cross-reactive memory responses elicited by previous exposure to other *Salmonella* serovars [42–44], or other Enterobacteriaceae, including those present in the normal gut microbiota [22, 45–47]. Several studies highlight the importance of the gut microbiota in modulating host immune responses to pathogens or to vaccination [45–47]. We have recently shown that vaccination against *S.* Typhi caused no alterations of the microbiota, however, individuals displaying early multiphasic CMI responses harbored more diverse communities than late responders [47]. We have initiated studies to identify the interplay between the host immune response, the microbiota and clinical outcome in volunteers challenged with wt *S.* Typhi. In addition to these acquired immune response differences, genetic determinants like HLA molecules can also be critical in defining the variation in immune responses. For example, the presence of the *HLA*-DRB1*04:05 allele was recently associated with protection against *S.* Typhi [48].

## Conclusions

In summary, we have provided the first direct evidence of an association between higher baseline levels of multifunctional *S.* Typhi-specific CD8+ T cells and protection, as well as delayed time to disease onset, in an oral challenge model with wt *S.* Typhi in humans. These studies also revealed some of the immunological responses associated with delayed time to disease onset and defined the homing characteristics of the *S.* Typhi-specific effector and memory CD8+ T cell populations. This information supports the performance of in depth CMI measurements to aid in the early selection of novel vaccine candidates for further development and evaluation in clinical trials.

## Additional file

10.1186/s12967-016-0819-7 Participant Demographic Characteristics. **Figure S1.** Baseline hematological parameters and *S.* *typhi*-specific responses. (a) Total white cell count (WCC) and absolute numbers of lymphocytes were assessed by routine blood hematology before challenge. Absolute numbers of CD3+, CD3+CD8+, CD3+CD4+ cells, and of CD3+CD8+ memory subsets (T_EM_, T_EMRA_ and T_CM_) were calculated using the percentages of positive cells obtained by flow cytometry analysis. Statistical analyses were performed using the Mann–Whitney test. (b) Individual representation of baseline CD8+ T_EM_ immune responses following stimulation with *S.* *typhi*-infected cells. PBMC isolated at baseline from each participant (TD n = 7, blue; NoTD n = 9, red) were stimulated for 18 h with *S.* *typhi*-infected B-EBV cells (circles) or *S.* *typhi*-infected blasts (triangles). After co-culture, cells were immunostained with a 14-color panel of mAbs and analyzed by follow cytometry as described in Methods. Each symbol represents the net percentage of positive CD8+ T_EM_ cells measured for CD107a, IFN-γ, TNF-α, MIP-1β, as indicated. Horizontal lines represent the median for each group. Statistical analyses were performed using the Mann–Whitney test. *p < 0.05; **p < 0.01. **Figure S2.** Absolute numbers of T cell subsets in circulation after challenge. Shown are the kinetics of various T cell subsets in representative participants from the TD and NoTD groups. One participant who did not meet diagnosis definition (NoTD) but showed the presence of *S.* *typhi* in blood by PCR assay is also represented. (a) Absolute numbers of CD3+ and CD3+CD8+cells following challenge were calculated using the percentages of positive cells obtained by flow cytometry analysis. (b) Absolute numbers of IFN-γ+ and CD107a expressing *S.* *Typhi*-specific CD8+ T_EM_ following challenge. **Figure S3.** Kinetics and amplitude of *S*. *Typhi*-specific CD8+ T cell responses after challenge for each participant. (a) Kinetics of IFN-γ production by CD8+ T_EM_ following stimulation with *S.* *Typhi*-infected B-EBV cells are presented for each participant. The day at which each of the TD participants was diagnosed is indicated after their id number. (b) Areas under the curve were measured around the time of diagnosis for each biomarker in CD8+ T_EM_, T_EMRA_ and T_CM_ subsets. Each bar represents mean ± SEM of area under the curve obtained after stimulation with *S.* *Typhi*-infected AEH cells, B-EBV cells and blasts. **Figure S4.** Homing potential of the dominant *S.* *Typhi*-specific CD8+ T_EM_ multifunctional populations at baseline. Flow cytometry data were analyzed using the FCOM function of Winlist to determine the proportion of all possible combinations of the six measured biomarkers to identify MF cells (i.e., positive for multiple biomarkers concomitantly). Each symbol represents the percentage of the different populations measured after stimulation with *S.* *Typhi*-infected cells (AEH cells [squares], B-EBV [circles] cells or blasts [triangles]) for each participant. (a) The percentages of single positive cells (1+) or of total MF cells (i.e., the sum of all cells concomitantly positive for 2 or more biomarkers) are represented for CD8+ T_EM_ integrin α_4_β_7_− and integrin α_4_β_7_+ cells. (b) Total MF cells were divided into four groups on the basis of the number of biomarkers they expressed. (C) Shown are the six major individual populations of MF in integrin α_4_β_7_− and integrin α_4_β_7_+ *S.* *Typhi*-specific CD8+ T_EM_ at baseline in all NoTD participants. Horizontal lines represent the median for each group. Statistical analyses were performed using mixed effects models to account for multiple observations per person. *p < 0.05, **p < 0.01, ***p < 0.001. **Figure S5.** Dominant populations and gut homing capabilities of MF *S.* *Typhi*-specific CD8+ T_EM_ responses in TD and NoTD participants after challenge. Flow cytometry data were analyzed using the FCOM function of Winlist to determine the proportion of all possible combinations of the six measured biomarkers to identify MF cells (i.e., positive for several biomarkers concomitantly). Percentages were measured at day 7 for NoTD participants and at 48 h after typhoid diagnosis for TD participants. Each symbol represents the percentage of the different populations measured after stimulation with *S.* *typhi*-infected cells (AEH cells [squares], B-EBV [circles] cells or blasts [triangles]) for each participant. (a) The percentages of single positive cells (1+) or of total MF cells (i.e., the sum of all cells concomitantly positive for two or more biomarkers) are represented for CD8+ T_EM_ integrin α_4_β_7_− and integrin α_4_β_7_ + cells. (b) Total MF cells were divided into four groups on the basis of the number of biomarkers they expressed. (c) The six major individual populations of MF in CD8+ T_EM_ are represented separately for *S.* *typhi*-specific integrin α_4_β_7_− (green) and integrin α_4_β_7_+ (purple) CD8+ T_EM_ in NoTD participants at day 7 post-challenge. Horizontal lines represent the median for each group. Statistical analyses were performed using mixed effects models to account for multiple observations per person. *p < 0.05, **p < 0.01, ***p < 0.001.
