# Supplementary material for: Salmonella Typhi-specific multifunctional CD8+ T cells play a dominant role in protection from typhoid fever in humans
Source: J Transl Med. 2016 Mar 1;14:62. doi: 10.1186/s12967-016-0819-7 (PMC4772330; doi:10.1186/s12967-016-0819-7)
Supplement: Supplementary file 1 — 10.1186/s12967-016-0819-7 Participant Demographic Characteristics. Figure S1. Baseline hematological parameters and S. typhi-specific responses. (a) Total white cell count (WCC) and absolute numbers of lymphocytes were assessed by routine blood hematology before challenge. Absolute numbers of CD3+, CD3+CD8+, CD3+CD4+ cells, and of CD3+CD8+ memory subsets (TEM, TEMRA and TCM) were calculated using the percentages of positive cells obtained by flow cytometry analysis. Statistical analyses were performed using the Mann–Whitney test. (b) Individual representation of baseline CD8+ TEM immune responses following stimulation with S. typhi-infected cells. PBMC isolated at baseline from each participant (TD n = 7, blue; NoTD n = 9, red) were stimulated for 18 h with S. typhi-infected B-EBV cells (circles) or S. typhi-infected blasts (triangles). After co-culture, cells were immunostained with a 14-color panel of mAbs and analyzed by follow cytometry as described in Methods. Each symbol represents the net percentage of positive CD8+ TEM cells measured for CD107a, IFN-γ, TNF-α, MIP-1β, as indicated. Horizontal lines represent the median for each group. Statistical analyses were performed using the Mann–Whitney test. *p < 0.05; **p < 0.01. Figure S2. Absolute numbers of T cell subsets in circulation after challenge. Shown are the kinetics of various T cell subsets in representative participants from the TD and NoTD groups. One participant who did not meet diagnosis definition (NoTD) but showed the presence of S. typhi in blood by PCR assay is also represented. (a) Absolute numbers of CD3+ and CD3+CD8+cells following challenge were calculated using the percentages of positive cells obtained by flow cytometry analysis. (b) Absolute numbers of IFN-γ+ and CD107a expressing S. Typhi-specific CD8+ TEM following challenge. Figure S3. Kinetics and amplitude of S. Typhi-specific CD8+ T cell responses after challenge for each participant. (a) Kinetics of IFN-γ production by CD8+ TEM foll [file 12967_2016_819_MOESM1_ESM.docx]

SUPPLEMENTAL TABLE AND FIGURES

| **Supplemental Table. Participant Demographic Characteristics** | |
| --- | --- |
| Characteristic | Participants |
| No. of participants challenged | 16 |
| Sex, male, No. (%) | 12 (75) |
| Age, y, mean ± SD (range) | 30.1 ± 10.5 (19.4-46.5) |
| Ethnicity, white, No. (%) | 15 (94) |
| SD, standard deviation. |  |

Supplemental Figure 1. Baseline hematological parameters and *S.* Typhi-specific responses. (a) Total white cell count (WCC) and absolute numbers of lymphocytes were assessed by routine blood hematology before challenge. Absolute numbers of CD3+, CD3+CD8+, CD3+CD4+ cells, and of CD3+CD8+ memory subsets (T_EM_, T_EMRA_ and T_CM_) were calculated using the percentages of positive cells obtained by flow cytometry analysis. Statistical analyses were performed using the Mann-Whitney test. (b) Individual representation of baseline CD8+ T_EM_ immune responses following stimulation with *S.* Typhi-infected cells. PBMC isolated at baseline from each participant (TD n=7, blue; NoTD n=9, red) were stimulated for 18h with *S.* Typhi-infected B-EBV cells (circles) or *S.* Typhi-infected blasts (triangles). After co-culture, cells were immunostained with a 14-color panel of mAbs and analyzed by follow cytometry as described in Methods. Each symbol represents the net percentage of positive CD8+ T_EM_ cells measured for CD107a, IFN-γ, TNF-α, MIP-1β, as indicated. Horizontal lines represent the median for each group. Statistical analyses were performed using the Mann-Whitney test. *p<0.05; **p<0.01

Supplemental Figure 2. Absolute numbers of T cell subsets in circulation after challenge. Shown are the kinetics of various T cell subsets in representative participants from the TD and NoTD groups. One participant who did not meet diagnosis definition (NoTD) but showed the presence of *S.* Typhi in blood by PCR assay is also represented. (a) Absolute numbers of CD3+ and CD3+CD8+ cells following challenge were calculated using the percentages of positive cells obtained by flow cytometry analysis. (b) Absolute numbers of IFN-γ+ and CD107a expressing *S.* Typhi-specific CD8+ T_EM_ following challenge.

Supplemental Figure 3. Kinetics and amplitude of *S*. Typhi-specific CD8+ T cell responses after challenge for each participant. **(a)** Kinetics of IFN-γ production by CD8+ T_EM_ following stimulation with *S.* Typhi-infected B-EBV cells are presented for each participant. The day at which each of the TD participants was diagnosed is indicated after their id number. (b) Areas under the curve were measured around the time of diagnosis for each biomarker in CD8+ T_EM_, T_EMRA_ and T_CM_ subsets. Each bar represents mean ± SEM of area under the curve obtained after stimulation with *S.* Typhi-infected AEH cells, B-EBV cells and blasts.

Supplemental Figure 4. Homing potential of the dominant *S.* Typhi-specific CD8+ T_EM_ multifunctional populations at baseline. Flow cytometry data were analyzed using the FCOM function of Winlist to determine the proportion of all possible combinations of the 6 measured biomarkers to identify MF cells (i.e., positive for multiple biomarkers concomitantly). Each symbol represents the percentage of the different populations measured after stimulation with *S.* Typhi-infected cells (AEH cells [squares], B-EBV [circles] cells or blasts [triangles]) for each participant. **(a)** The percentages of single positive cells (1+) or of total MF cells (i.e., the sum of all cells concomitantly positive for 2 or more biomarkers) are represented for CD8+ T_EM_ integrin α_4_β_7_- and integrin α_4_β_7_+ cells. (b) Total MF cells were divided into 4 groups on the basis of the number of biomarkers they expressed. (**C**) Shown are the 6 major individual populations of MF in integrin α_4_β_7_- and integrin α_4_β_7_+ *S.* Typhi-specific CD8+ T_EM_ at baseline in all NoTD participants. Horizontal lines represent the median for each group. Statistical analyses were performed using mixed effects models to account for multiple observations per person. *p<0.05, **p<0.01, ***p<0.001.

Supplemental Figure 5. Dominant populations and gut homing capabilities of MF *S.* Typhi-specific CD8+ T_EM_ responses in TD and NoTD participants after challenge. Flow cytometry data were analyzed using the FCOM function of Winlist to determine the proportion of all possible combinations of the 6 measured biomarkers to identify MF cells (i.e., positive for several biomarkers concomitantly). Percentages were measured at day 7 for NoTD participants and at 48 hours after typhoid diagnosis for TD participants. Each symbol represents the percentage of the different populations measured after stimulation with *S.* Typhi-infected cells (AEH cells [squares], B-EBV [circles] cells or blasts [triangles]) for each participant. (a) The percentages of single positive cells (1+) or of total MF cells (i.e., the sum of all cells concomitantly positive for 2 or more biomarkers) are represented for CD8+ T_EM_ integrin α_4_β_7_- and integrin α_4_β_7_+ cells. (b) Total MF cells were divided into 4 groups on the basis of the number of biomarkers they expressed. (c) The 6 major individual populations of MF in CD8+ T_EM_ are represented separately for *S.* Typhi-specific integrin α_4_β_7_- (green) and integrin α_4_β_7_+ (purple) CD8+ T_EM_ in NoTD participants at day 7 post-challenge. Horizontal lines represent the median for each group. Statistical analyses were performed using mixed effects models to account for multiple observations per person. *p<0.05, **p<0.01, ***p<0.001.
